# Acute Sterol O-Acyltransferase 2 (SOAT2) Knockdown Rapidly Mobilizes Hepatic Cholesterol for Fecal Excretion

**DOI:** 10.1371/journal.pone.0098953

**Published:** 2014-06-05

**Authors:** Stephanie M. Marshall, Anthony D. Gromovsky, Kathryn L. Kelley, Matthew A. Davis, Martha D. Wilson, Richard G. Lee, Rosanne M. Crooke, Mark J. Graham, Lawrence L. Rudel, J. Mark Brown, Ryan E. Temel

**Affiliations:** 1 Department of Pathology, Section on Lipid Sciences, Wake Forest University School of Medicine, Winston-Salem, North Carolina, United States of America; 2 Cardiovascular Group, Antisense Drug Discovery, Isis Pharmaceuticals, Carlsbad, California, United States of America; 3 Department of Cellular and Molecular Medicine, Cleveland Clinic Foundation – Lerner Research Institute, Cleveland, Ohio, United States of America; 4 Saha Cardiovascular Research Center, University of Kentucky, Lexington, Kentucky, United States of America; University of Bari & Consorzio Mario Negri Sud, Italy

## Abstract

The primary risk factor for atherosclerotic cardiovascular disease is LDL cholesterol, which can be reduced by increasing cholesterol excretion from the body. Fecal cholesterol excretion can be driven by a hepatobiliary as well as a non-biliary pathway known as transintestinal cholesterol efflux (TICE). We previously showed that chronic knockdown of the hepatic cholesterol esterifying enzyme sterol O-acyltransferase 2 (SOAT2) increased fecal cholesterol loss via TICE. To elucidate the initial events that stimulate TICE, C57Bl/6 mice were fed a high cholesterol diet to induce hepatic cholesterol accumulation and were then treated for 1 or 2 weeks with an antisense oligonucleotide targeting SOAT2. Within 2 weeks of hepatic SOAT2 knockdown (SOAT2^HKD^), the concentration of cholesteryl ester in the liver was reduced by 70% without a reciprocal increase in hepatic free cholesterol. The rapid mobilization of hepatic cholesterol stores resulted in a ∼2-fold increase in fecal neutral sterol loss but no change in biliary cholesterol concentration. Acute SOAT2^HKD^ increased plasma cholesterol carried primarily in lipoproteins enriched in apoB and apoE. Collectively, our data suggest that acutely reducing SOAT2 causes hepatic cholesterol to be swiftly mobilized and packaged onto nascent lipoproteins that feed cholesterol into the TICE pathway for fecal excretion.

## Introduction

Despite advances in treatment and prevention, cardiovascular disease remains the number one killer of Americans [1]. High blood concentrations of LDL cholesterol (LDLc) lead to the development of atherosclerosis, which is the principal cause of the majority of clinical cardiovascular events [1]. By inhibiting cholesterol synthesis and consequently increasing LDL clearance from the blood, statins have the ability to significantly reduce LDLc and have been shown to reduce the risk of cardiovascular disease by as much as 44% [2]. However, statin treatment is not always effective at lowering LDLc to the recommended target level and can cause side effects such as myopathy and elevated liver enzymes [3]. Therefore, it is important to develop other treatments that will reduce LDLc or modulate LDL atherogenicity. A promising treatment option is the inhibition of sterol O-acyl transferase 2 (SOAT2) also termed acyl-CoA:cholesterol *O*-acyl transferase 2 (ACAT2). SOAT2 is a transmembrane-associated enzyme localized to the endoplasmic reticulum of hepatocytes and enterocytes and catalyzes the transfer of the fatty acid from long chain acyl CoA to the 3′ hydroxyl group of cholesterol [4], [5]. The cholesteryl esters (CE) produced by SOAT2 can be packaged into nascent, apoB-containing chylomicrons and VLDL or stored in cytosolic neutral lipid droplets [6]. We have previously shown that *in vitro* a systematic increase in SOAT2 expression resulted in increased CE secretion in apoB-containing lipoproteins [7]. Mice with whole-body or intestine-specific knockout of Soat2 have reduced cholesterol absorption [8]–[10] due to an inability to efficiently package cholesterol as CE into chylomicrons [11]. Deficiency of Soat2 in liver results in the secretion of VLDL that are depleted of CE [12] thus resulting in a significant reduction in plasma VLDLc concentration [10], [13]–[16]. Mice with whole-body or liver-specific disruption of Soat2 often do not display a change in plasma LDLc [10], [14], [17] because of the ability of lecithin-cholesterol acyltransferase (LCAT) to form CE on plasma LDL [12], [14], [18]. However, regardless of plasma LDLc concentration, Soat2 deficiency significantly reduces atherosclerosis development in Ldlr-/- and Apoe-/- mice [13]–[15], [17]. The reduced atherogenicity of LDL from Soat2 deficient mice appears to be caused in part by SOAT2-derived cholesteryl oleate depletion that decreases LDL binding to proteoglycans [13].

Similar to targeted gene deletion, inhibition of function or disruption of expression of SOAT2 by pharmacological means causes major alterations in cholesterol homeostasis and atherosclerosis development. Treatment of Apoe-/- mice with the SOAT2 selective inhibitor pyripyropene A caused reductions in cholesterol absorption, plasma VLDLc and LDLc concentration, cholesteryl oleate content of apoB-containing lipoproteins, and atherosclerosis progression [19]. By using an antisense oligonucleotide targeting Soat2 mRNA (SOAT2 ASO), SOAT2 expression was knocked down in a liver-specific manner resulting in decreased LDL cholesteryl oleate and diminished aortic atherosclerosis development [20].

It was anticipated that hepatic SOAT2 knockdown (SOAT2^HKD^) would cause free cholesterol (FC) to accumulate in the liver since cholesterol absorption would be normal but the hepatocytes would be unable to esterify any excess cholesterol delivered by chylomicrons. In spite of unaltered cholesterol absorption and a near absence of SOAT2 expression and activity in liver, hepatic FC concentration was normal in apoB100 only, Ldlr-/- mice with SOAT2^HKD^ [21]. To presumably protect the liver from FC toxicity, there was a 2-fold increase in fecal cholesterol excretion in SOAT2^HKD^ mice. Since mice treated with SOAT2 ASO had no change in biliary cholesterol secretion and normal cholesterol absorption, we hypothesized that the increased fecal cholesterol excretion was the result of increased transintestinal cholesterol efflux (TICE), a process by which cholesterol is secreted into the lumen of the small intestine after being delivered through plasma to the enterocytes [22], [23]. To determine whether the liver of SOAT2 ASO-treated mice was producing a lipoprotein that was preferentially targeted for clearance by the small intestine, isolated liver perfusion was conducted on mice that had been radiolabeled with [^3^H]cholesterol and treated with control or SOAT2 ASO. The radiolabeled perfusate, which carried almost 100% of the cholesterol on VLDL, was then injected into control and SOAT2 ASO treated mice. After 6 hr, 2–3 fold more [^3^H]cholesterol from the SOAT2^HKD^ perfusate compared to the control perfusate had accumulated in the lumen and wall of the proximal small intestine. From this result we concluded that the VLDL secreted from the SOAT2^HKD^ liver was preferentially targeted to the small intestine [21].

In the current study, we wanted to determine the initial changes that occur in cholesterol metabolism when SOAT2 expression is knocked down in liver. To increase the likelihood of stimulating cholesterol excretion via the hepatobiliary and/or TICE pathways, C57BL/6 mice were fed a high cholesterol diet (0.2%; wt/wt) for 6 weeks to induce hepatic CE accumulation. After hepatic cholesterol loading, the mice were treated with control or SOAT2 ASO for 1–2 weeks. Acute treatment with SOAT2 ASO rapidly reduced hepatic SOAT2 expression and CE concentration but had no significant impact on FC in the liver. Similar to chronic SOAT2^HKD^, acute SOAT2^HKD^ did not alter biliary cholesterol concentration but almost doubled fecal cholesterol excretion therefore indicating that the TICE pathway was stimulated. The increased plasma concentration of FC, apoB, and apoE suggests that the liver of SOAT2^HKD^ may be producing lipoproteins that preferentially feed cholesterol into the TICE pathway.

## Materials and Methods

### Mice

Female C57BL/6N wild-type mice were maintained on standard rodent diet. At 6–8 weeks of age, the mice were switched to a semisynthetic low-fat, high-cholesterol diet (10% of energy as palm oil-enriched fat, 0.2% cholesterol w/w) and maintained on this diet for the remainder of the study. After 6 weeks of high-cholesterol diet feeding, mice were injected intraperitoneally biweekly with 25 mg/kg of either non-targeting ASO (Control ASO-ISIS 353512 (5′-TCCCATTTCAGGAGACCTGG-3′) [24] or ASO directed against murine SOAT2 (SOAT2 ASO-ISIS 217006 (5′-TTCGGAAATGTTGCACCTCC-3′), as previously described [20], [21], [24]. After 1 or 2 weeks of treatment, mice were fasted for 4 hr and anesthetized with ketamine/xylazine (120/20 mg/kg, intramuscular injection). Bile was collected from the gallbladder. Blood was collected by heart puncture, and euthanasia was achieved by exanguination. Following a whole body flush with saline, the liver and small intestine were collected and snap frozen in liquid nitrogen. All mice were maintained in an American Association for Accreditation of Laboratory Animal Care-approved animal facility under protocols approved by the Institutional Animal Care and Use Committee at Wake Forest University School of Medicine.

### Immunoblotting of Tissue Proteins and Lipoprotein Apolipoproteins

Liver microsomes were prepared as previously described [25]. Briefly, liver samples were homogenized in SOAT2 homogenization buffer (0.25 M sucrose, 1 mM EDTA, 0.1 M K_2_HPO_4_) in the presence of protease inhibitor cocktail (Sigma) and then centrifuged to remove cellular debris. The supernatant was centrifuged with added protease inhibitor at 100,000×g for 1 hour to isolate the microsomal fraction. Western blots of whole liver lysates for ABCA1, TGH1, and liver microsomes for SOAT2 were conducted as previously described [21], [24]. For apolipoprotein analysis, an equal volume of plasma from each mouse within a treatment group was pooled. 200 µL of pooled plasma for groups treated with diet only, SOAT2 ASO for 1 week, SOAT2 ASO for 2 weeks and control ASO for 2 weeks and 150 µL of pooled plasma for the group treated with control ASO for 1 week were separated on a Superose 6 10/300 GL column (GE Healthcare) at a flow rate of 0.4 ml/min and fractions were collected at defined intervals. An equal volume within lipoprotein fractions from the groups diet only, SOAT2 ASO for 1 week, SOAT2 ASO for 2 weeks and control ASO for 2 weeks and 25% more volume of the fractions from the group treated with control ASO for 1 week were mixed with 5X SDS sample buffer and separated on a NuPAGE Novex 4–12% Bis Tris Midi Gel using 1X NuPAGE MOPS SDS running buffer (Invitrogen). The proteins were transferred to a nitrocellulose membrane, which was subsequently blocked with 5% (w/v) non-fat dried milk dissolved in wash buffer. The membrane was incubated overnight at 4°C with one or more of the following antibodies: rabbit anti-human apoAI (Biodesign), goat polyclonal to human apoB (Academy Biomedical) or rabbit polyclonal to rat apoE (provided by Dr. Joachim Herz, UT Southwestern Medical Center). After washing, the blots were exposed to secondary antibodies against goat or rabbit IgG conjugated to horseradish peroxidase (Sigma), and the probed proteins were visualized with ECL reagent (PerkinElmer) and exposure to Blue X-Ray Film (Phenix).

### Quantitative Real-Time PCR (qPCR)

RNA extraction and qPCR were conducted as previously described on individual tissue samples (n = 5 per group) [8]. Cyclophilin was used as an internal control, and mRNA expression levels were calculated based on ΔΔ-CT method. Messenger RNA levels for each gene represent the amount relative to that of WT mice treated with control ASO, which was arbitrarily standardized to 1. Primer sequences used for qPCR are available on request.

### Plasma Concentration and Distribution of Cholesterol

Plasma total cholesterol concentration and lipoprotein cholesterol distributions were determined as described previously [26]. Plasma free cholesterol concentration was determined using the Free Cholesterol E reagent (Wako). For determination of the free cholesterol to total cholesterol ratio (FC/TC) in lipoprotein classes, an equal volume of plasma from each mouse within a treatment group was pooled. 200 µL of pooled plasma for the groups treated with diet only, SOAT2 ASO for 1 week, SOAT2 ASO for 2 weeks and control ASO for 2 weeks and 150 µL of pooled plasma for the group treated with control ASO for 1 week were separated on a Superose 6 10/300 GL column (GE Healthcare) at a flow rate of 0.4 ml/min and fractions were collected at defined intervals. A known volume of each interval was Bligh-Dyer extracted in tubes containing 5-alpha cholestane as an internal standard [27]. Once the collected chloroform was dried down the extract was re-suspended in hexane and analyzed by gas chromatography (GC) [7]. After analysis for free cholesterol concentration the hexane was collected and dried down so the extract could be saponified for total cholesterol analysis by GC.

### Liver and Gallbladder Lipid Measurements

Lipid concentrations in the liver and the gallbladder bile were determined as described previously [8], [28], [29].

### Analysis of Fecal Neutral Sterol Excretion

Immediately following the first ASO injection, mice were individually housed in wire bottom cages to collect feces for neutral sterol analysis. After 72 hr, the mice were administered a second dose of the respective ASO and transferred to clean wire bottom cages for a second collection of feces. After 96 hr in this setting, the mice were fasted for 4 hr, euthanized, and bile, plasma, liver, and small intestine were collected for analysis. A second set of mice received their first week of ASO doses in normal caging. After their third ASO dose, the mice were individually housed in wire bottom cages and feces were collected for 72 hr. These mice then received another ASO dose and were transferred to a clean wire bottom cages in order to collect feces for 96 hr. Tissue and fluid samples were then collected from these mice as described above. Fecal neutral sterol excretion was measured as described previously [8].

### Statistical Analysis

Data are expressed as the mean ± standard error of the mean (SEM) and were analyzed using multivariate analysis of variance (ANOVA), followed by Student's t tests for post hoc analysis. Differences were considered significant at p<0.05. All analyses were performed using JMP version 5.0.12 (SAS Institute; Cary, NC) software, unless otherwise specified.

## Results

### SOAT2 knockdown rapidly reduces hepatic cholesteryl ester stores

**Figure 1:**
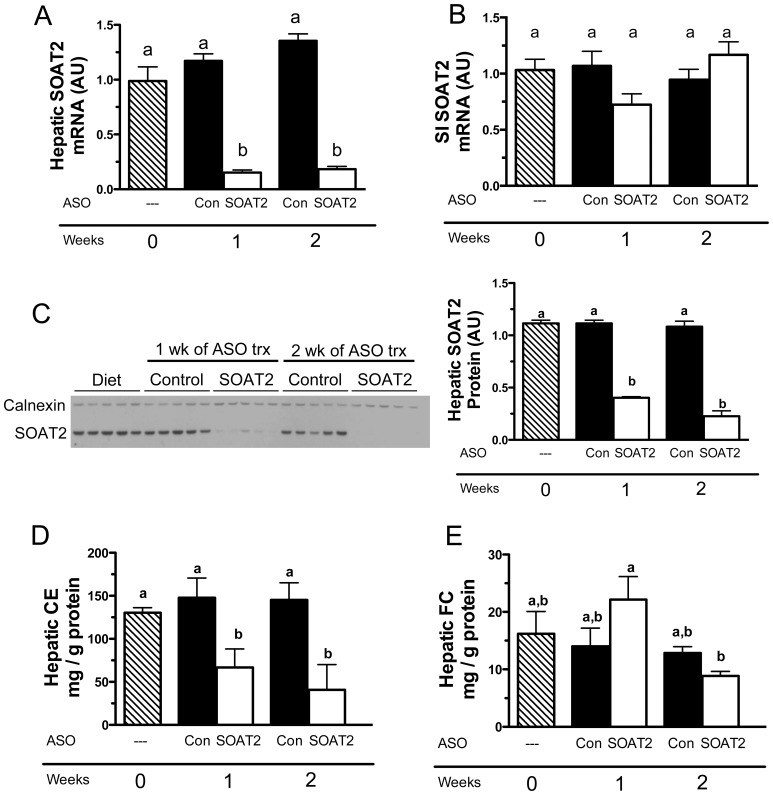
Hepatic SOAT2 knockdown rapidly reduces cholesteryl ester levels in liver. After consuming a high cholesterol diet for 6 weeks, mice were either euthanized to collect baseline samples or were continued on diet and administered for 1 or 2 weeks control ASO or SOAT2 ASO. Quantitation of SOAT2 mRNA in liver (A) and proximal third of small intestine (B) were conducted by real-time PCR using individual RNA samples (n = 5 per treatment group). (C) Western blot analysis was used to detect hepatic SOAT2 protein expression in liver microsomes. To compare the relative expression of SOAT2 between treatment groups, the band intensity of SOAT2 was normalized to that of calnexin and quantification of protein levels was determined by densitometry (C). Fasting livers were collected and analyzed for the concentration of cholesteryl ester (CE) (D) and free cholesterol (FC) (E). All hepatic lipid values were normalized to the protein content of the extracted tissue, and represent the means ± SEM (4–6 mice per treatment group). Bars not sharing a common letter differ significantly (p<0.05).

Female C57BL/6N wild-type mice were fed a high-cholesterol diet (0.2% w/w) for 6 weeks to induce hepatic cholesteryl ester (CE) accumulation, and were then treated for 1 or 2 weeks with either control ASO or an ASO targeting SOAT2. Within 1 week of administration of SOAT2 ASO versus control ASO, hepatic SOAT2 mRNA was reduced by >85% (Figure 1A). SOAT2 ASO treatment did not significantly alter intestinal SOAT2 mRNA expression (Figure 1B). Knockdown of SOAT2 mRNA for 1 week reduced hepatic SOAT2 protein by 67% (Figure 1C) and hepatic CE by 55% (Figure 1D). SOAT2 protein and CE in liver were decreased by 80% and 72%, respectively, in mice treated for 2 weeks with SOAT2 ASO compared to control ASO. Hepatic free cholesterol (FC) was not significantly different in mice administered SOAT2 ASO versus control ASO for 1 or 2 weeks (Figure 1E). However, there was a 60% reduction in hepatic FC in mice treated with SOAT2 ASO for 2 weeks versus 1 week (Figure 1E).

### Acute hepatic SOAT2 knockdown increases fecal cholesterol excretion in mice without altering biliary lipids

**Figure 2:**
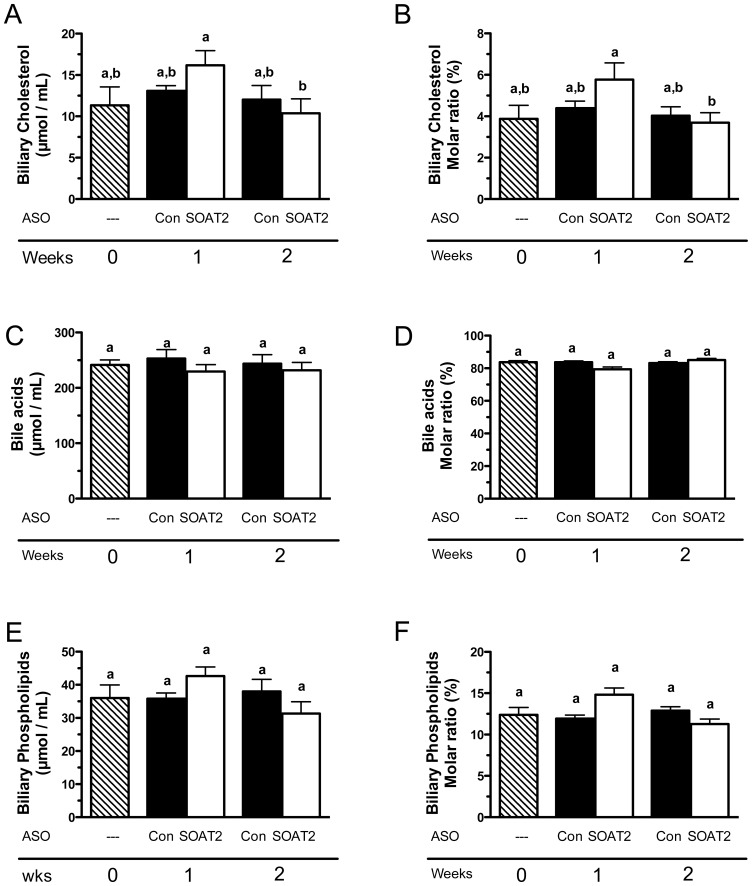
Impact of acute hepatic SOAT2 knockdown on the concentration and molar ratio of lipids in gallbladder bile. Mice were treated as described in the legend for Figure 1. Gallbladder bile was collected and analyzed for the concentration of cholesterol (A), bile acids (B), and phospholipids (C). To calculate the molar ratio of cholesterol (D), bile acids (E), and phospholipids (F), the concentration of each biliary lipid was divided by the sum of the concentrations of the three biliary lipids. Data represent the means ± SEM (n = 5–6 mice per treatment group). Bars not sharing a common letter differ significantly (p<0.05).

**Figure 3:**
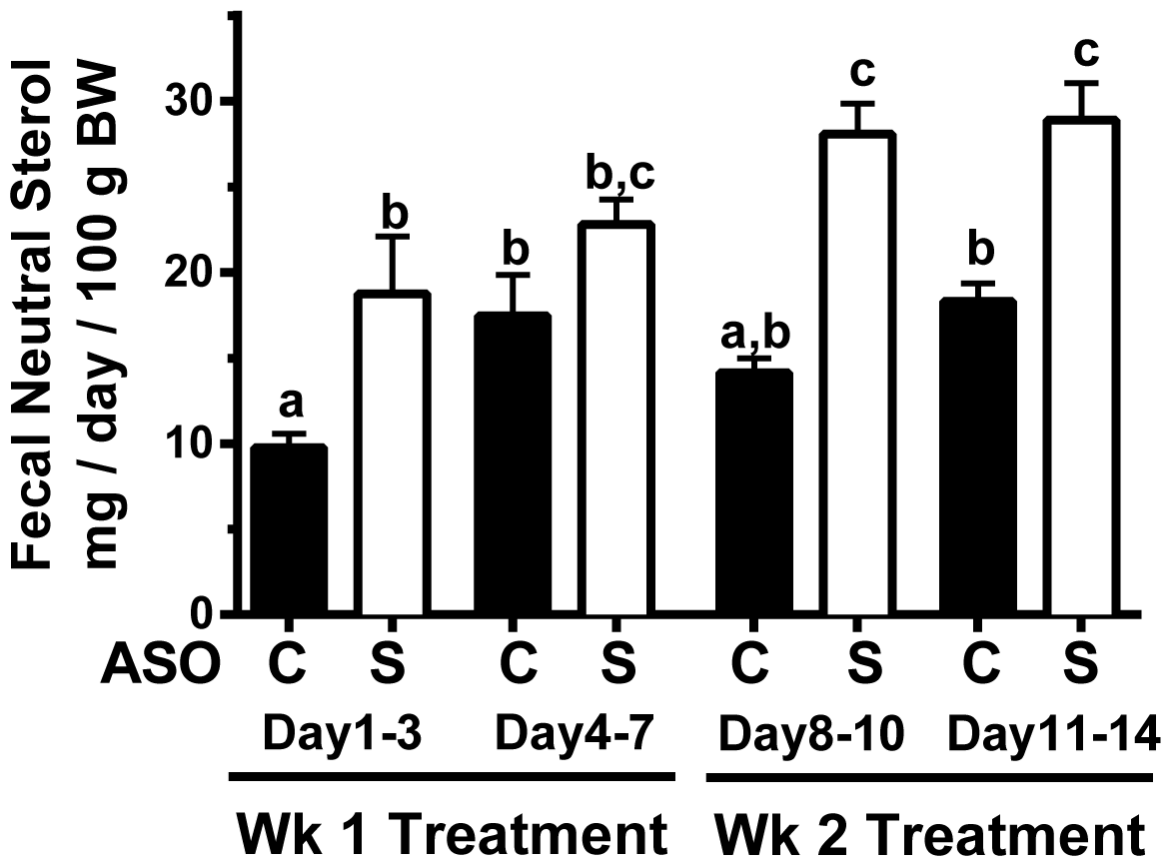
Fecal neutral sterol excretion in mice with acute knockdown of SOAT2 in liver. Mice were fed the high cholesterol diet for 6 weeks and then continued on the same diet during 1 or 2 weeks of ASO treatment. Immediately following the first injection of control ASO [C] or SOAT2 ASO [S], mice were individually housed in wire bottom cages to collect feces for neutral sterol analysis. After 72 hours (Day 1–3), the mice were administered a second dose of ASO and transferred to clean wire bottom cages for a second collection of feces. After 96 hours (Day 4–7) in this setting, the mice were fasted for 4 hours and euthanized for fluid and tissue collection. A second set of mice received their first week of ASO doses in normal caging. After the third ASO dose, the mice were individually housed in wire bottom cages and feces were collected for 72 hours (Day 8–10). These mice then received another ASO dose and were transferred to a clean wire bottom cages in order to collect feces for 96 hrs (Day 11–14). Tissue and fluid samples were then collected from these mice as described above. Neutral sterol was extracted from the feces and quantitated using gas-liquid chromatography. Data represent the means ± SEM (n = 5–6 mice per treatment group). Bars not sharing a common letter differ significantly (p<0.05).

We next investigated whether the rapid and dramatic reduction in CE caused by hepatic SOAT2 knockdown (SOAT2^HKD^) resulted in increased cholesterol movement into the bile. When biliary lipid levels of gallbladder bile were measured, biliary cholesterol concentration (Figure 2A) was not significantly increased in mice treated with SOAT2 ASO compared with control ASO or diet alone. In contrast, mice dosed with SOAT2 ASO for 2 weeks versus 1 week did display a significant 36% decrease in biliary cholesterol concentration (Figure 2A). To normalize for differences caused by the bile being concentrated while stored in the gallbladder, the molar ratio of cholesterol in bile was calculated. Acute SOAT2^HKD^ had almost identical qualitative effects on the molar ratio (Figure 2B) and concentration (Figure 2A) of cholesterol in bile. Excess cholesterol can also be eliminated from the liver by conversion into bile acids. However, similar to cholesterol, SOAT2^HKD^ did not increase bile acid concentration (Figure 2C) or molar ratio (Figure 2D). Biliary phospholipids solubilize newly secreted biliary cholesterol; yet, biliary phospholipid concentration (Figure 2E) and molar ratio (Figure 2F) also were unchanged upon SOAT2^HKD^. Although there were no major alterations in biliary lipids when hepatic CE was rapidly depleted, fecal neutral sterol excretion was increased 92% within 72 hr of the first SOAT2 ASO injection (Figure 3). With the exception of the 96 hr period following the second ASO injection (Day 4-7), output of neutral sterol into the feces was significantly greater at each time point in mice treated with SOAT2 ASO compared to control ASO (Figure 3).

### Acute hepatic SOAT2 knockdown transiently increases plasma total cholesterol and doubles plasma free cholesterol

**Figure 4:**
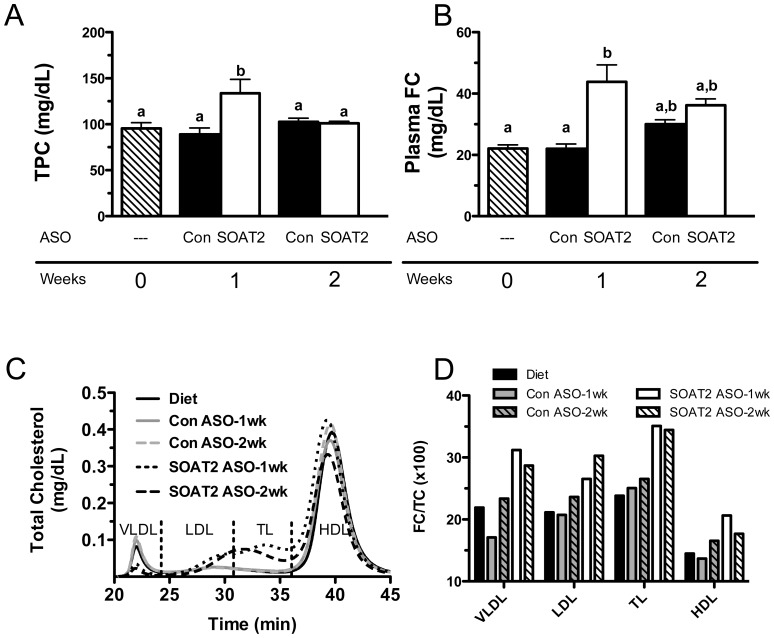
Plasma cholesterol concentration and distribution in mice with acute hepatic SOAT2 knockdown. After eating a high cholesterol diet for 6 weeks, mice were either euthanized to collect baseline samples or were continued on diet and administered for 1 or 2 weeks control ASO or SOAT2 ASO. Fasting plasma was isolated from 4–6 mice per treatment group and analyzed for total cholesterol (TPC) (A) and free cholesterol (FC) (B) concentration. Data represent the means ± SEM and bars not sharing a common letter differ significantly (p<0.05). Pooled plasma from 4–5 mice per treatment group was separated by gel filtration chromatography (C) and fractions containing VLDL, LDL, transition lipoproteins [TL], and HDL (D) were collected and analyzed by gas-liquid chromatography to determine the FC to TC ratio in each lipoprotein fraction.

Plasma total cholesterol (TC) (Figure 4A) and plasma FC (Figure 4B) were both significantly increased after one week of SOAT2^HKD^, suggesting that a proportion of the hepatic CE stores was rapidly secreted into the plasma. After 2 weeks of treatment, plasma concentrations of TC and FC were no longer different between mice receiving SOAT2 ASO and control ASO. In order to determine which lipoprotein classes were being impacted by acute SOAT2^HKD^, TC distribution on plasma lipoproteins was measured. As observed previously [21], VLDL-TC was reduced upon hepatic SOAT2 knockdown (Figure 4C). SOAT2 ASO treatment for 1 and 2 weeks increased TC in LDL and small LDL/large HDL, which are referred to in this work as transition lipoproteins (TL). This “transition” lipoprotein simply refers to the fact that these lipoproteins elute between the canonical LDL and HDL peaks following size exclusion chromatography. HDL-TC was similar for mice treated with SOAT2 ASO compared to control ASO for either 1 or 2 weeks (Figure 4C). However, SOAT2^HKD^ for 2 weeks compared to 1 week appeared to substantially decrease HDL-TC (Figure 4C). To define the lipoprotein classes enriched in FC, VLDL, LDL, TL, and HDL was isolated from plasma using gel filtration chromatography (Figure 4C) and the ratio of FC to TC in the fractions was determined. SOAT2^HKD^ appeared to increase the FC to TC ratio in all of the lipoprotein classes at both times of observation, but this ratio was most prominently increased in the VLDL and TL fractions (Figure 4D).

### Apolipoprotein content of plasma lipoproteins following acute hepatic SOAT2 knockdown

**Figure 5:**
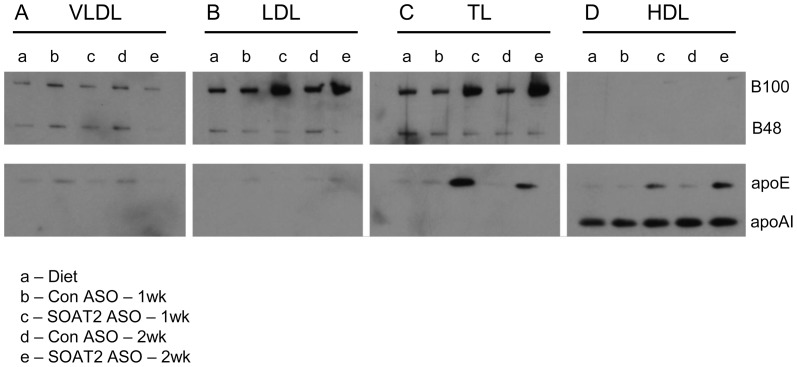
Apolipoprotein content of isolated plasma lipoproteins following acute hepatic SOAT2 knockdown. Fasting plasma was isolated from mice treated with diet only (a), control ASO for 1 week (b), SOAT2 ASO for 1 week (c), Control ASO for 2 weeks (d), and SOAT2 ASO for 2 weeks (e). Pooled plasma was separated by gel filtration chromatography and fractions containing VLDL (A), LDL (B), transition lipoproteins [TL] (C), and HDL (D) were collected. An equal volume within a lipoprotein fraction was loaded onto a 4–12% polyacrylamide-SDS gel. Following SDS-PAGE, the lipoprotein fractions were immunoblotted to determine the relative abundance of apoB, apoE and apoAI.

Analysis of the relative apolipoprotein abundance in the VLDL, LDL, TL, and HDL fractions showed the major apolipoprotein species present among the lipoprotein fractions. Compared to mice receiving control ASO, mice with SOAT2^HKD^ had a reduction in apoB100 and apoB48 in VLDL (Figure 5A). By contrast, apoB100 abundance was increased in the LDL (Figure 5B) and TL (Figure 5C) fractions of mice treated for 1 and 2 weeks with SOAT2 ASO versus control ASO. Similar to other murine models where TICE appears to be increased [30], mice with acute hepatic SOAT2 knockdown had increased apoE abundance in TL and HDL (Figure 5C and 5D). Consistent with the relatively stable HDL-TC concentrations, apoAI abundance in HDL was similar among the different treatment groups.

### Hepatic expression of genes involved in cholesterol homeostasis in mice with acute knockdown of SOAT2

**Figure 6:**
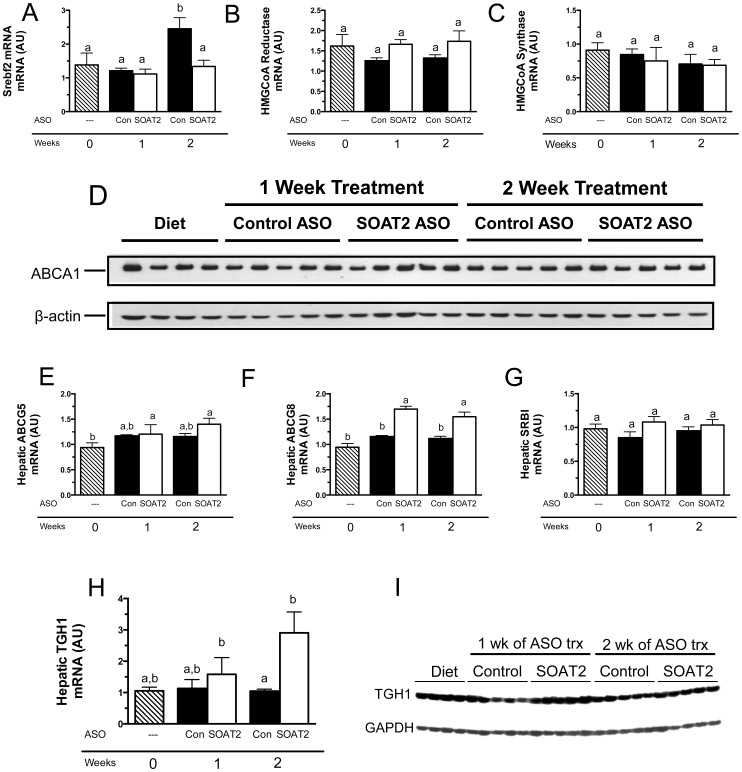
Hepatic expression of genes involved in cholesterol homeostasis in mice with acute hepatic SOAT2 knockdown. Mice were treated as described in Figure 3 and fasting liver was collected. Quantitation of hepatic Srebf2 (A), HMG-CoA Reductase (B) and HMG-CoA Synthase (C), SR-BI (D), ABCG5 (E), ABCG8 (F) were conducted by real-time PCR using individual RNA samples (n = 5). Data represent the means ± SEM, and bars not sharing a common letter differ significantly (p<0.05). Western blot analysis was used to quantify hepatic ABCA1 protein expression for individual liver samples (G). Quantitation of hepatic TGH (H) was conducted by real-time PCR using individual RNA samples (n = 5). Western blot analysis was used to assess hepatic TGH1 protein expression for individual liver samples (I).

Although FC concentrations were unchanged when comparing mice treated with SOAT2 ASO and control ASO (Figure 1E), it was possible that acute SOAT2^HKD^ was increasing a regulatory pool of FC that was inactivating sterol response binding element protein 2 (SREBP2) or stimulating liver X receptor (LXR) thus altering hepatic expression of genes involved in cholesterol homeostasis. A reduction in cholesterol synthesis could potentially compensate for an increase in FC caused by hepatic SOAT2 depletion. However, the mRNA abundance of HMG-CoA synthase, HMG-CoA reductase, and SREBP2 were not altered in mice treated with the SOAT2 ASO for 1 or 2 weeks (Figure 6A-C). By upregulating the expression of the LXR target genes ABCG5, ABCG8 and ATP binding cassette transporter A1 (ABCA1), excess FC could have been effluxed from the livers of SOAT2^HKD^ mice to bile or HDL, respectively. However, unlike our previous study with chronic SOAT2^HKD^ [21], hepatic ABCA1 protein did not appear to increase with acute SOAT2^HKD^ (Figure 6D). SOAT2 ASO treatment did not alter the hepatic expression of ABCG5 mRNA (Figure 6E) but did slightly increase hepatic ABCG8 mRNA (Figure 6F). Furthermore, the hepatic mRNA expression of the HDL receptor SR-BI was unchanged by SOAT2 ASO treatment (Figure 6G). Interestingly, an enzyme that has been shown to hydrolyze triglyceride and cholesteryl ester, triglyceride hydrolase 1 (TGH1), was slightly increased at both the mRNA and protein level with SOAT2 ASO treatment (Figure 6H-I) [31].

### Intestinal expression of genes involved in cholesterol metabolism are not altered by SOAT2 ASO treatment

**Figure 7:**
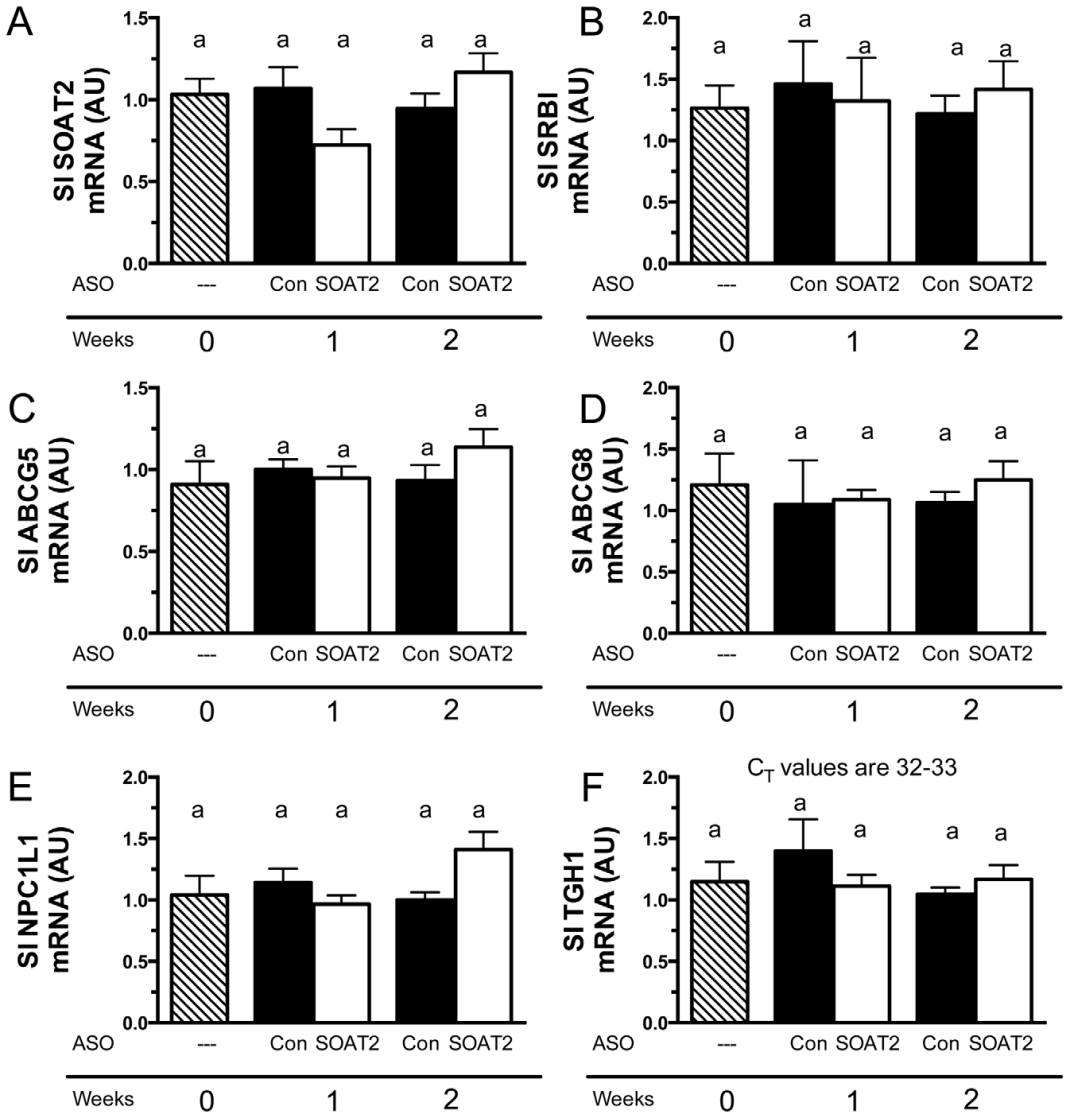
Intestinal expression of genes involved in cholesterol homeostasis in mice with acute hepatic SOAT2 knockdown. Mice were treated as described in the legend for Figure 1. Quantitation of intestinal SOAT2 (A), SRBI (B), ABCG5 (C), ABCG8 (D), NPC1L1 (E) and TGH1 (F) mRNA were conducted by real-time PCR using individual RNA samples (n = 5 per treatment group). Data represent the means ± SEM (4–6 mice per treatment group). Bars not sharing a common letter differ significantly (p<0.05).

Given that SOAT2 ASO treatment promotes rapid flux of liver-derived cholesterol to the small intestine, we interrogated the expression of genes involved in cholesterol metabolism in the proximal small intestine where TICE is most active. Interestingly, SOAT2 ASO treatment failed to alter the expression of SOAT2, SR-BI, ABCG5, ABCG8, NPC1L1, or TGH1. These results suggest that SOAT2 ASO treatment has divergent effects on gene expression in the liver (Figure 6) and small intestine (Figure 7).

## Discussion

Chronic hepatic knockdown of SOAT2 expression appears to stimulate TICE resulting in increased cholesterol excretion and decreased LDLc concentration [21]. In the current study, we sought to determine the initial changes that occur in cholesterol homeostasis when SOAT2 expression is knocked down in liver. In mice that had been previously fed a high-cholesterol diet to induce hepatic CE accumulation, treatment with SOAT2 ASO for 1-2 weeks caused 1) knockdown of hepatic SOAT2 expression and consequently rapid depletion of CE from the liver, 2) increased plasma FC carried on apoB- and apoE-containing lipoproteins and 3) elevated fecal neutral sterol excretion without major perturbations in biliary cholesterol. Since our current results are consistent with those caused by chronic SOAT2 knockdown [21], we conclude that cholesterol liberated from the liver due to acute hepatic SOAT2 knockdown is rapidly mobilized onto lipoproteins that feed into the TICE pathway.

Acute treatment with SOAT2 ASO caused a rapid and dramatic reduction in hepatic SOAT2 expression (Figure 1A and 1C) and a concomitant breakdown of CE stores in the liver (Figure 1D). It is reasonable to assume that upon SOAT^HKD^ in cholesterol-fed mice, the hepatic cholesterol esterification and hydrolysis cycle was unbalanced resulting in rapid turnover of stored CE. Neutral lipid hydrolases such as TGH-1/CES3, HSL, and neutral cholesteryl ester hydrolase may have been responsible for the breakdown of CE (Figure 6H–I) [31], [32]. In particular, the hepatic expression of TGH1 was stimulated slightly by SOAT2 ASO treatment (Figure 6H–I). It is also possible that the rapid depletion of hepatic CE was driven by autophagy, which has been shown to play a major role in CE turnover in macrophage foam cells [33], [34].

Although consistent with our previous studies of SOAT2 knockdown and knockout in the liver [10], [21], it was quite surprising that hepatic FC concentration was not significantly altered in mice acutely treated with SOAT2 ASO versus control ASO (Figure 1D). The absorption of cholesterol should have been normal in SOAT^HKD^ mice [21] resulting in the delivery of cholesterol-rich chylomicron remnants to the liver. The majority of the cholesterol coming from the chylomicron remnants and other plasma lipoproteins should have remained unesterified in hepatocytes with SOAT2 knockdown. Moreover, the livers of SOAT^HKD^ mice faced the additional burden of FC originating from the rapid turnover of CE in the intracellular lipid droplets. Nevertheless, hepatocytes with SOAT2 knockdown were able to adapt to the new sources of FC and maintain cellular FC at a proper level. Reducing cholesterol synthesis in the liver could have offset the increase in hepatic FC caused by acute SOAT2 knockdown. However, mRNA expression of the master transcriptional regulator of cholesterol biosynthesis Srebf2 and its target genes HMGCoA reductase and HMGCoA synthase were not changed in mice treated acutely with SOAT2 ASO (Figure 6A–C). Excess FC could have also been eliminated from the liver by secretion directly into bile or conversion to bile acids. Yet acute treatment with SOAT2 ASO compared to control ASO did not significantly alter the concentration of cholesterol (Figure 2A) and bile acids (Figure 2B) in gallbladder bile. Like our previous findings that gallbladder cholesterol and bile acid levels were unaltered in mice with chronic SOAT^HKD^ [21], our current results indicate that acute knockdown of SOAT2 in liver does not drive FC towards secretion into bile or conversion into bile acids. However, since biliary lipids were only measured in gallbladder bile collected after 1 and 2 weeks of SOAT2 ASO treatment, it is possible that biliary secretion of cholesterol and bile acids was significantly increased prior to our two time points for gallbladder bile sampling.

Based upon the finding that fecal neutral sterol excretion was significantly increased in mice acutely treated with SOAT2 ASO compared to control ASO (Figure 3), it appears SOAT2 knockdown caused the liver to funnel excess hepatic cholesterol into the feces for elimination. Since biliary cholesterol concentration was unchanged (Figure 2A), it seems likely that the surplus hepatic cholesterol was being directed into the TICE pathway. This conclusion is consistent with our previous study showing that chronic SOAT^HKD^ caused a doubling of fecal neutral sterol excretion and an elevation in the trafficking of liver-derived cholesterol through the blood to the small intestine [21]. In the case of both chronic and acute SOAT^HKD^, excess hepatic cholesterol is presumably secreted into the blood after being packaged on nascent lipoproteins. The liver has the capacity to create both HDL and VLDL and thus either class of lipoprotein could be involved in moving cholesterol to the small intestine for TICE. With acute SOAT^HKD^, there appeared to be no change in the protein expression of hepatic ABCA1 (Figure 6D), which is necessary for formation of nascent HDL by the liver [35] and consequently FC efflux. Plasma HDL-TC concentration of mice appeared to be unchanged after 1 week and slightly reduced following 2 weeks of SOAT2 ASO treatment (Figure 4C). The ratio of FC to TC on plasma HDL was modestly increased with SOAT^HKD^ but was much lower than that on other lipoprotein classes (Figure 4D) due presumably to the ability of LCAT to preferentially convert HDL FC to CE. Although we cannot conclude from our study whether HDL was involved in moving hepatic cholesterol to the TICE pathway, a recent study from Vrins et al indicated that TICE was unaltered in Abca1 deficient mice, which have very low HDL levels [36].

With acute SOAT^HKD^, excess hepatic cholesterol could have also been packaged into VLDL or smaller apoB-containing lipoproteins for delivery through the plasma to the small intestine. This scenario is supported by our previous work that showed the liver of mice treated chronically with SOAT2 ASO versus control ASO secreted nascent VLDL that appeared to deliver more cholesterol to the TICE pathway [21]. In addition, Le May et al recently reported that TICE can be increased by eliminating PCSK9-dependent degradation of intestinal LDLR [37]. However, intestinal uptake of cholesterol from apoB-containing lipoprotein must not solely rely on LDLR since mice deficient in LDLR have normal or increased levels of TICE [21], [37]. In our current study, plasma from acute SOAT^HKD^ mice contained VLDL, LDL, and TL that were enriched in FC (Figure 4D) indicating the liver was secreting a greater amount of FC on apoB-containing lipoproteins. Treatment with SOAT2 ASO compared to control ASO appeared to cause a reduction in both TC and apoB100 associated with VLDL (Figure 4C and 5A), which could be interpreted as increased VLDL clearance by the small intestine. In contrast, elevated amounts of TC and apoB100 were found in the LDL and TL fraction of plasma from acute SOAT^HKD^ mice (Figure 4C, 5B–C). It is possible that SOAT2 knockdown caused the liver to produce smaller, more FC-rich LDL that feed cholesterol into TICE. Alternatively, the accumulation of TC and apoB100 in plasma LDL and TL could reflect reduced hepatic LDLR expression due SOAT2 knockdown altering hepatic cholesterol homeostasis.

Acute SOAT^HKD^ caused a substantial increase in the level of TC and apoE found in the plasma TL fraction (Figure 4C and 5C). In addition, TL from SOAT^HKD^ mice had the highest ratio of FC to TC (∼30%) when compared to other lipoproteins classes isolated from the plasma of mice treated with either SOAT2 ASO or control ASO (Figure 4D). It is tempting to speculate that apoE-rich lipoproteins act as a sink for cholesterol that is destined for the TICE pathway. Certainly, apoE-rich TL are found to accumulate in the plasma of LXR-agonist treated mice and Niemann-Pick C1-like 1 hepatic transgenic mice (NPC1L1 Tg), which have been reported to have increased TICE [28], [30], [37]–[39]. However, we have recently discovered that NPC1L1 Tg mice deficient in apoE have normal fecal neutral sterol excretion (Temel and Brown, unpublished data) therefore indicating that apoE may not be required for efficient cholesterol delivery to the TICE pathway.

Our studies of acute and chronic SOAT2 hepatic knockdown suggest that SOAT2 inhibition can increase TICE. In addition, it has been shown that small molecule inhibition of SOAT2 decreases cholesterol absorption [19]. Taken together these data suggest that inhibiting both intestinal and hepatic SOAT2 should maximally stimulate fecal neutral sterol loss given the dual effects of promoting TICE and limiting intestinal cholesterol re-absorption. Thus, SOAT2 inhibition could lower CVD risk by not only decreasing the amount of pro-atherogenic cholesteryl oleate on LDL [13], [17], [19], [20] but also reducing the availability of cholesterol for incorporation into LDL. Research is ongoing to create highly selective SOAT2-specific small molecule inhibitors that could hold promise for improved prevention and treatment of CVD in the post-statin era.
